# An Anatomic Single-Suture Trans-osseous Technique for the Repair of Acute Achilles Tendon Ruptures

**DOI:** 10.7759/cureus.19092

**Published:** 2021-10-28

**Authors:** Panagiotis V Samelis, Evangelos Triantafyllou, Dimitrios Artsitas, Charikleia Komari, Stefania Nikolaou

**Affiliations:** 1 2nd Orthopaedic Department, Apostolos Pavlos Trauma Hospital, Athens, GRC

**Keywords:** fixation, suture, transosseous, rupture, tendon, achilles

## Abstract

Several surgical methods for the treatment of acute Achilles tendon ruptures have been described. Whether open or percutaneous, these methods may be subdivided into two categories: all-soft-tissue procedures or procedures with stabilization of the Achilles tendon directly on the os calcaneum. The former comprise end-to-end suturing of the tendon stumps, the latter include additional stabilization of the sutures on the calcaneus either with bone anchors or by means of trans-osseous sutures. We describe a new, simple, anatomic, trans-osseous suture technique to stabilize the Achilles tendon on the calcaneus.

## Introduction

The Achilles tendon (AT) is the strongest and largest tendon of the body [1]. It transmits the forceful contraction of the gastrocnemius-soleus complex to the calcaneus [1]. Its normal strength and anatomy are of utmost importance for activities of daily living, work, or sports participation.

AT pathology ranges clinically from mild chronic pain to acute rupture [2]. Frequently, it is a sports-related injury, secondary to sudden and powerful contraction of the gastrocnemius-soleus muscles. This contraction is either concentric, during ankle plantar flexion, or eccentric, during ankle dorsiflexion. It is usually seen in young to middle-aged male adults. In most cases, AT rupture is located 2-6 cm proximal to the calcaneal insertion. Less frequently, a non-osseous avulsion of the AT insertion is observed, known as Achilles sleeve avulsion [3]. Rarely, a pure osseous avulsion of the AT from the calcaneal tuberosity may occur. Quinolone intake or corticosteroid treatment may predispose to AT rupture [4].

Acute AT ruptures should be treated immediately, within 48 hours from patient admission [5]. If untreated, granulation tissue is formed within a week, compromising conservative treatment [2]. Further delay of treatment leads to irreversible contracture of the gastrocnemius-soleus muscle and permanent loss of muscle length and strength [2].

Several methods have been described to treat acute AT ruptures. Open surgery, minimally invasive surgery, or conservative treatment with functional rehabilitation have been implemented [2,5]. Despite the general conception for sports-related injuries, that surgical treatment would yield better and more predictable results, several reports and meta-analyses conclude that operative and conservative treatment lead to equal long-term results. Furthermore, open surgery seems to bear a higher risk for wound-related complications [5,6].

Surgical treatment of AT ruptures may be subdivided into two main categories: all-soft-tissue procedures or procedures which involve direct stabilization of the AT on the calcaneal bone. Bony stabilization may be accomplished either with bone anchors or by means of trans-osseous calcaneal sutures.

A simple trans-osseous stitch through the calcaneal tuberosity is presented. A single suture is passed through the distal AT stump and through an osseous tunnel at the level of the anatomical AT insertion and continued as a Krakow stitch to the proximal AT stump [7]. Tying the suture ends approximates the tendon stumps and creates a central pillar to accommodate the degenerated fraying fascicles of the ruptured AT.

## Technical report

**Figure 1:**
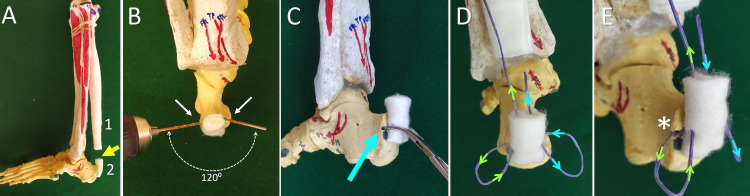
The transosseous single-suture technique for the repair of Achilles tendon rupture A. An Achilles tendon (AT) rupture model (yellow arrow). The proximal (1) and the distal (2) AT stump. B. Two converging osseous tunnels at an angle of 120 degrees are drilled on either side of the calcaneus at the level of the AT insertion. C. A towel forceps is used to convert the two osseous tunnels to a single curved tunnel (blue arrow). D. The insertion (blue arrows) and exit (green arrows) of the suture from the distal AT stump. E. The calcaneal tunnel at the level of AT insertion (asterisk)

The technique is described on a plastic lower limb skeleton model. The steps to repair the AT are shown in Figure 1:

Two osseous tunnels on either side of the AT insertion are drilled with the 2.5 mm drill, forming an angle of approximately 120⁰ from each other. A towel forceps are used to unite these tunnels. A curved osseous tunnel at the level of the AT insertion is created.

One strong (Nr. 1.5), non-absorbable suture enters the distal AT stump intralesionally and exits at the level of the AT insertion. The suture is propagated into the calcaneal tunnel, exits this tunnel at the other side of the calcaneus, is re-inserted into the proximal AT stump, and exits at the ruptured end of the AT.

**Figure 2:**
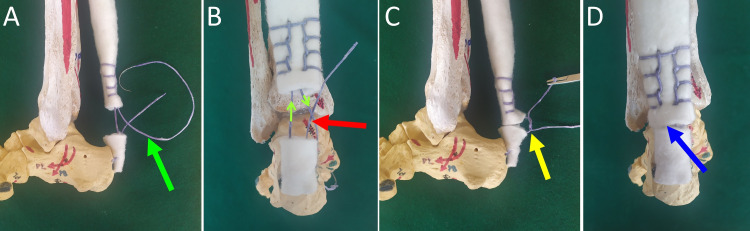
Achilles tendon rupture repair with an anatomic transosseous single suture technique A. Completion of the suture technique (green arrow). B. The Krakow stitch is shown (green arrows). The two suture ends are approximated (red arrow). C. The suture ends are tied. The tension of the repair is controlled  to avoid over-tensioning or shortening of the Achilles tendon (yellow arrow). D. Achilles tendon repair, dorsal view (blue arrow)

The same suture continues as a Krakow stitch on both sides of the proximal AT stump. Four or five locking loops, 5 mm apart, are placed along the medial and lateral side of the proximal AT stump. The suture exits the proximal AT stump intralesionally, opposite to its other end at the proximal stump insertion. The free ends of the suture are tied together (Figure 2).

In the clinical setting, the patient is placed in the prone position. Starting from the calcaneal insertion, the AT is exposed through a 10-12 cm medially placed longitudinal incision. Care is taken to preserve the paratenon. Before tying the suture, it is recommended to pull the Krakow stitch in order to remove any slack and to pre-tension the contracted gastrocnemius-soleus muscle. This decreases the gap between the tendon stumps and the tension of the repair [2]. The first knot between the free ends of the suture is gradually tensioned and temporarily secured using a mosquito clamp. The Thompson test is elicited. If negative, the knot is permanently locked with additional throws. In case of marked AT degeneration, the fraying ends of the AT lesion are approximated around the central suture using any appropriate suture technique the surgeon prefers. The paratenon and the skin are closed in layers. The ankle is splinted in 20⁰ of flexion.

## Discussion

**Figure 3:**
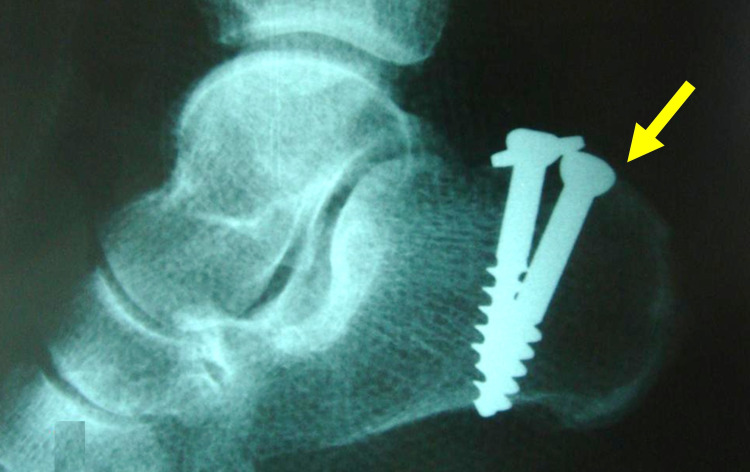
Avulsion fracture of the achilles tendon from the calcaneal tuberosity in a 63-year-old female. Uneventful healing of the fracture after internal fixation with two lag screws (arrow).

**Figure 4:**
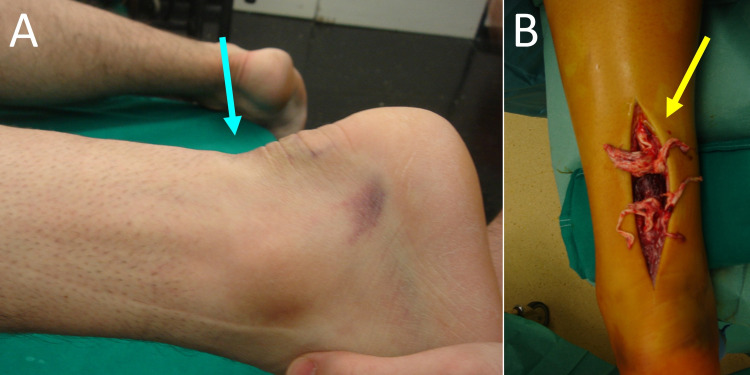
Acute left achilles tendon rupture in a 40-year-old male. A. The typical dorsal gap of the skin at the level of the achilles tendon rupture (blue arrow). B. The "spaghetti-like" appearance of the ruptured achilles tendon ends, indicating pre-existing degeneration of the tendon substance (yellow arrow).

Pure osseous avulsions of the AT from the calcaneal tuberosity usually heal after lag screw fixation (Figure 3). However, it has been shown that, in acute tendon ruptures some degree of chronic tendon degeneration is almost always present [8]. Very often, the stumps of the ruptured AT have a spaghetti-like appearance (Figure 4). The poor quality of the ruptured AT ends bears a significant risk for treatment failure.

In the past, modified Kessler or Bunnel stitches had been used for end-to-end repair of acute AT ruptures. Since its description in 1986, the Krakow stitch is the widely adopted method for the repair of this lesion [7]. However, only the proximal part of the Krakow stitch provides a strong tissue purchase, since it may be extended considerably on the healthy proximal part of the AT. The same is not true for the distal part of this stitch. In most cases, marked fraying of the distal part of the tendon mandates stabilization on the calcaneus using bone anchors or trans-osseous sutures. Osseous stabilization of the AT using suture-loaded bone anchors or trans-osseous sutures is effective to deal with the weak grip of sutures on the degenerated or small distal AT stump, or in case of AT sleeve or osseous avulsions [6,9-12].

Yang et al. described a trans-osseous suture technique to treat AT sleeve avulsion injuries. They drilled three osseous tunnels through the calcaneal tuberosity, at the level of the AT insertion. The tunnels had a cephalad-caudal direction. Two Nr. 2 non-absorbable sutures were whip-stitched on either side of the AT. One free end of each suture was passed through the central calcaneal tunnel, the other free ends were passed through each lateral tunnel. The AT was pulled to its anatomic calcaneal insertion and the sutures were tied [3].

Longo et al. described a five-incision technique (including a Cincinnati incision at the AT calcaneal insertion) to treat AT avulsions. They used a Bunnell-type technique with a pair of strong non-absorbable sutures to pull the tendon distally. The tendon was fixed to the calcaneal insertion using two bone anchors. Additional stitches were used to reinforce the repair [6].

Zhang et al. inserted one suture-loaded anchor to the AT calcaneal footprint. An end-to-end repair with two Kessler-type stitches was performed [12].

Yin et al. described the panda rope bridge technique to treat acute AT ruptures [11]. They inserted two suture-loaded bone anchors on either side of the calcaneus, distal to the AT insertion. The free ends of the sutures were passed below the leg fascia up to the level of the muscle-tendon junction of the AT. Two Krakow stitches were placed on either side of the AT and tied at this level. Additional sutures were used to re-approximate the degenerated AT fibers at the rupture site [11].

Isik et al. described a two-suture technique. One trans-osseous suture was inserted percutaneously through the calcaneal body, distal to the AT insertion. The free ends of the trans-osseous suture were passed subcutaneously and exited at a longitudinal midline skin incision. The second suture was a bilateral Krakow-stitch at the proximal AT stump. The free ends of the sutures on either side of the AT were tied together. One additional suture-loaded bone anchor was placed at the calcaneus to reinforce the midportion of the repair [10].

Sundararajan et al. used a trans-osseous technique, which was described for rotator cuff repair, to reattach the AT back to its footprint. They did not extend any stitches proximally on the AT to counteract gastrocnemius-soleus contraction. Weight-bearing was allowed only after the eighth postoperative week [13].

Fanter et al. described a trans-calcaneal suture-button technique to reattach AT avulsions. A Krakow stitch was inserted on either side of the AT. The free ends of the sutures were loaded on a suture button. Under fluoroscopic control, a trans-calcaneal osseous tunnel was drilled between the AT footprint and the plantar surface of the calcaneus, anterior to the lateral calcaneal process. The loaded suture button was passed through the trans-calcaneal osseous tunnel and deployed on the undersurface of the calcaneus. After tensioning the suture button, a double row bridging technique was used to reattach the AT on its footprint [14].

Pavlou et al. created two trans-calcaneal tunnels. The free ends of a whip-stitch of the proximal stump of the AT are tied on the undersurface of the calcaneus [15].

All these techniques are interesting, but they are either complicated, time-consuming, or expensive. Some demand special surgical equipment. We describe a simple trans-osseous single-stitch technique to stabilize the proximal AT stump on its calcaneal insertion. Additional sutures may be implemented to obtain end-to-end repair and to augment the original stitch. This single-suture technique bears less risk for knot loosening or AT overtightening and subsequent AT shortening. Furthermore, the trans-osseous tunnel is drilled directly at the anatomic AT insertion, thus providing an anatomic repair. This technique is also useful in the treatment of AT sleeve avulsions or chronic ruptures. In such cases, we additionally perform a mild rejuvenation of the AT footprint, to promote faster healing. In all cases, an immediate postoperative splint and rehabilitation protocols are recommended as usual.

## Conclusions

A trans-osseous suture technique to stabilize the proximal part of the ruptured AT on the calcaneus is described. This technique has several advantages. It is simple and cheap since no special surgical equipment or bone anchors are required. The calcaneal osseous tunnel is placed at the level of the AT footprint, thus providing an anatomic repair. Gradual tightening of the suture allows control of the gastrocnemius-soleus-AT tension to avoid over-tightening and shortening of the AT. The suture creates a central pillar to approximate the fraying degenerated tendon ends. This technique can be used to treat chronic AT ruptures and AT sleeve avulsions as well. Additional sutures may be placed to reinforce the original trans-osseous stitch. Postoperative rehabilitation protocols are recommended as usual.
